# DBatVir: the database of bat-associated viruses

**DOI:** 10.1093/database/bau021

**Published:** 2014-03-18

**Authors:** Lihong Chen, Bo Liu, Jian Yang, Qi Jin

**Affiliations:** MOH Key Laboratory of Systems Biology of Pathogens, Institute of Pathogen Biology, Chinese Academy of Medical Sciences & Peking Union Medical College, Beijing, China

## Abstract

Emerging infectious diseases remain a significant threat to public health. Most emerging infectious disease agents in humans are of zoonotic origin. Bats are important reservoir hosts of many highly lethal zoonotic viruses and have been implicated in numerous emerging infectious disease events in recent years. It is essential to enhance our knowledge and understanding of the genetic diversity of the bat-associated viruses to prevent future outbreaks. To facilitate further research, we constructed the database of bat-associated viruses (DBatVir). Known viral sequences detected in bat samples were manually collected and curated, along with the related metadata, such as the sampling time, location, bat species and specimen type. Additional information concerning the bats, including common names, diet type, geographic distribution and phylogeny were integrated into the database to bridge the gap between virologists and zoologists. The database currently covers >4100 bat-associated animal viruses of 23 viral families detected from 196 bat species in 69 countries worldwide. It provides an overview and snapshot of the current research regarding bat-associated viruses, which is essential now that the field is rapidly expanding. With a user-friendly interface and integrated online bioinformatics tools, DBatVir provides a convenient and powerful platform for virologists and zoologists to analyze the virome diversity of bats, as well as for epidemiologists and public health researchers to monitor and track current and future bat-related infectious diseases.

**Database URL:** http://www.mgc.ac.cn/DBatVir/

## Introduction

Emerging infectious diseases have remained a major threat to public health during the past decades (1). Zoonotic diseases, or zoonoses, are diseases that are transmissible from animals to humans under natural conditions. Because ∼60% of all emerging infectious disease agents in humans are of zoonotic origin (1, 2), the study of animal diseases and their emerging potential has become increasingly important.

Bats are one of the most successful and diverse mammalian orders on the earth (3). Furthermore, >1200 bat species provide an unparalleled exhibition of variations on the mammalian theme and a broad lesson in biology (4, 5). In recent years, bats have gained significant notoriety after being implicated in numerous emerging infectious disease events, including the severe acute respiratory syndrome (SARS) outbreak 10 years ago and the current Middle East respiratory syndrome endemic (6–8). Moreover, bats have been suggested to be important reservoir hosts of many highly lethal zoonotic viruses that can cross species barriers to infect humans and other domestic or wild mammals, including rabies virus, Ebola virus, Marburg virus, Hendra virus and Nipah virus (9–11). Their ability to fly and social life history enable efficient virus maintenance, evolution and spread. Therefore, it is essential to enhance our knowledge and understanding of the genetic diversity of bat-associated viruses to prevent future outbreaks.

In recent years, next-generation sequencing methodologies have been used in a number of metagenomics studies dedicated to assessing the virome of bats, and these studies have revealed many known and novel viruses in bat samples (12–23). However, the most valuable information, such as the sampling time, location, bats species and specimen type, is available only sporadically in related literature or individual sequence records. Obtaining comprehensive information on bat-associated viruses becomes a formidable task. Therefore, we developed the database of bat-associated viruses (DBatVir), a comprehensive, up-to-date and well-curated repository of bat-associated animal viruses.

## Database construction

Molecular methods are now commonly used in the diagnosis and functional analyses of viruses; thus, DBatVir is built as a sequence-centric database. To retrieve all known sequences of bat-associated animal viruses from the public domain, we performed exhaustive searches in both the PubMed and Nucleotide databases of the National Center for Biotechnology Information (NCBI) using the keyword ‘(bat OR bats) AND (virus OR viruses)’. The related GenBank records were then downloaded and parsed using in-house BioPerl scripts to generate readable profiles for further expert review (24). Sequences of phages, insect and plant viruses, as well as samples derived from animals other than bats, were manually excluded. The associated metadata of each sequence, such as the sampling time, location, bat species, specimen type (e.g. feces, blood or tissues) and viral detection method (e.g. polymerase chain reaction or metagenomics), were further extracted from the related literature (if available) or the original GenBank records. Taxonomic information of all viruses and bats were retrieved from the Taxonomy database of NCBI. Additional information concerning the viruses, such as genome organization, average size and virion illustrations, and the bats, including common names, diet type and geographic distribution, were further collected from the ViralZone database (25) and the ICUN Red List (www.iucnredlist.org), respectively. The detailed phylogenetic relationships between the different bats, which were available from a previous study (26), were also carefully integrated into the database.

All aforementioned information was stored in a well-structured MySQL relational database, which is accessible through a series of Perl CGI scripts to dynamically generate the content for the foreground Apache web server. To provide a highly intuitive and responsive user interface, the ExtJS cross-browser JavaScript library (http://www.sencha.com/) was used to build desktop-like web pages. The standalone NCBI Basic Local Alignment Search Tool (BLAST) program was integrated into DBatVir web interface to allow users to perform sequence similarity searches in the database (27). The MUSCLE and FastTree programs were used for the development of online services of multiple sequence alignment and phylogenetic tree construction, respectively (28, 29). The jsPhyloSVG library was also included for the visualization of the phylogenetic trees on the web page (30).

## Database description and utility

### Web interface

**Figure 1.**
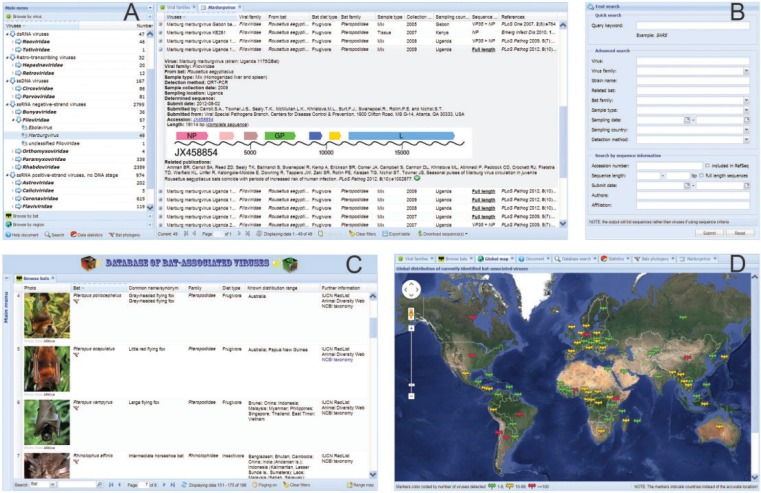
The web interface of DBatVir. (**A**) Multifunctional menu panel (left) and main content panel showing an information table of viruses with one line expanded (right). (**B**) Text search form enables both quick and advanced queries. (**C**) Collapsed menu panel (left vertical bar) and main content panel showing the bat-related information with a simple search engine on the bottom toolbar (right). (**D**) Main content panel showing the global distribution map with markers color-coded by number of bat-associated viruses currently detected. The content panel contains multiple different tabs (see above tab title cards).

The major utilities of the database, including database browsing/searching and live data statistics, as well as a detailed help document, are highlighted on the homepage by clickable icons with direct links to the respective main page. To offer a user-friendly interface, all information from the database is presented in a single desktop-like main page. A multifunctional menu panel is provided on the left side of the main page for easy navigation (Figure 1A), and this panel can be collapsed into a clickable vertical bar to maximize the visible section of the content panel on the right side (Figure 1C). The menu panel includes several submenus for users to browse the data by categories of viruses, bats or regions (see below). The content panel can handle multiple independent pages as different tabs in the panel, which behaves like an Excel workbook that contains multiple sheets (Figure 1D). Any new information requested by the users is presented in an individual tab in the content panel to avoid the unnecessary refresh of entire main page. In addition, the previously viewed content pages are hidden as inactive tabs rather than closed; thus, users can easily return to any previous content by clicking on the respective tab title to reactive it instantly without any redundant data reload.

**Figure 2.**
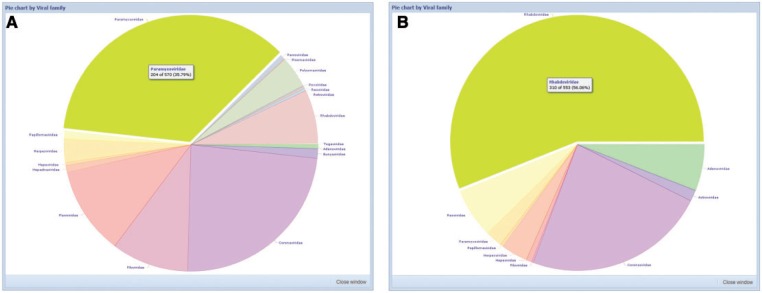
Statistical pie charts available for easy online analyses. (**A**) Viral family distribution of 570 viruses detected from bats in Africa. (**B**) Viral family distribution of 553 viruses detected from bats in Europe.

The quality of web interface is considered one of the most important aspects of a good database. We built a highly responsive and intuitive user interface by advanced JavaScript programming to provide users with the look and feel of a desktop application rather than a traditional web page. For example, all information tables are fully sortable and filterable with a single click on the column title, and each column is also movable and scalable (or hidden) by dragging and dropping it on the title. In addition, with a single click on the column title, a statistical pie chart of the selected information is available for easy online analyses (Figure 2). These features that were previously available only in stand-alone applications will undoubtedly provide high performance and an improved user experience.

### Browse the database

Users can browse the database content by the categories of viruses or bats using the submenus with the respective taxonomic tree in the menu panel. The hierarchical taxonomic trees show virus/bat families by default for brevity (Figure 1A). Each branch of the trees can be further expanded to show genera or species with a single click. Direct links to individual tabs in the content panel are given for each branch of the trees in the submenus. The main content panel presents a uniform grid for each tab that includes brief information on the viruses (species and family), specimens (sample type, collection date and sampling country), associated bats (species, family and diet type), determined sequences and related literature (exemplified in the right panel of Figure 1A). Each line of the table is expandable with a double click to show additional information, such as virus detection method, detailed sampling location, GenBank accessions and length of related sequences. Furthermore, a clickable linear map is offered for each complete viral sequence to highlight the genomic organization of the virus (Figure 1A). Handy buttons are provided on the bottom toolbar to export the grid as local Excel tables or to download the related sequences in FASTA format for further offline analyses. Browsing the data by virus and bat taxonomic categories is valuable to understand the host range of viruses and virome diversity of bats. For instance, from current data, the most widely distributed virus among bats is coronavirus, which has been detected from all the 10 bat families studied to date (Table 1). The bat family *Vespertilionidae* has been involved in 20 of the 23 virus families detected in bats so far (Table 1). It is reasonable given the fact that *Vespertilionidae* is the biggest family of bats containing >40 genera.

**Table 1.** Distribution of bat-associated animal viruses by virus and bat families (as of January 2014)

Virus family	Bat family^a^										Sum
	*Emballonuridae*	*Megadermatidae*	*Molossidae*	*Mormoopidae*	*Nycteridae*	*Phyllostomidae*	*Pteropodidae*	*Rhinolophidae*	*Rhinopomatidae*	*Vespertilionidae*	
*Adenoviridae*						1	5	8		51	65
*Astroviridae*	32						1	27		141	201
*Bunyaviridae*			12		1	3	3	15		2	36
*Caliciviridae*								5			5
*Circoviridae*			1				11	20		20	52
*Coronaviridae*	1	2	19	9	3	46	79	137	3	240	539
*Filoviridae*							36	1		1	38
*Flaviviridae*	6		16	1		11	51	12		6	103
*Hepadnaviridae*						5		5		10	20
*Hepeviridae*						1		2		4	7
*Herpesviridae*						1	19	4		23	47
*Orthomyxoviridae*						4					4
*Papillomaviridae*							9			3	12
*Paramyxoviridae*	1		1	7		23	262	13		23	330
*Parvoviridae*						8	4	24		43	79
*Picornaviridae*							1	3		12	16
*Polyomaviridae*		1	15	2		6	7			2	33
*Poxviridae*							1			1	2
*Reoviridae*			2				7	2		32	43
*Retroviridae*		1					4	6		1	12
*Rhabdoviridae*	6		360			196	77	8		1590	2237
*Totiviridae*										1	1
Total	46	4	426	19	4	305	577	292	3	2206	

^a^Viruses lacking related bat taxonomic information are excluded.

To provide an intuitive overview of the geographic distribution of current data, a global map with markers color-coded by number of bat-associated viruses detected in each country is also available (Figure 1D). Moreover, a submenu with a geographic category organized by continents and countries is provided in the menu panel for convenient regional bat-associated virus analyses. Each country/continent name offers a direct link to the respective tab in the content panel showing detailed information of the bat-associated viruses detected in corresponding region. It is particularly helpful to investigate the potential distribution bias of bat-associated viruses in different regions. For example, to date, 570 and 553 bat-associated viruses were detected from 19 counties of Africa and 17 countries of Europe, respectively. Though the general data are similar between the two continents, 18 virus families are found in bats from Africa, with *Paramyxoviridae* as the most predominant family (36%), whereas only 10 virus families are reported in bats from Europe and the principal family is *Rhabdoviridae* (56%) (Figure 2).

### Search the database

Usually, searching the database would be a more efficient way to find the required information than browsing the database. DBatVir provides a powerful search engine for users to extract information from the database quickly through three different ways: (i) text search (for viruses), (ii) BLAST sequence similarity search and (iii) bat-related information search.

The text search enables extracting virus information using any querying keywords. For a quick start, a single entry that is instantly familiar to users of common Internet search engines is offered. In addition, a configurable query form is available for advanced users to perform customized complex searches of all information in the database (Figure 1B). The search results are displayed in an individual tab in the content panel with explicit tables of bat-associated viruses matching the query. The result table is a high-performance grid as aforementioned, which enables instant sorting, filtering and table/sequences downloading, as well as easy online statistical analyses on the query output. Thus, the text search utility is not only useful to quickly extract required information but also valuable to make specialized analyses on any customized subset of the data from the database. For example, both feces and swabs are widely investigated samples of bats, and >500 viruses were detected from either type of samples worldwide. However, the viruses detected from swabs involve 17 virus families, whereas those from feces are limited to 11 families. It is noteworthy that swabs are a generic sample type that include fecal swabs, rectal swabs, oral swabs, nasal swabs and others. But many studies mixed different kinds of swabs (or even different types of samples) for easy virus detection. So researchers should be more specific in any cross-study comparative analysis and more cautious in follow-up data interpretation.

Sequence similarity searches within the database are available using the configurable BLAST submission form in the search panel. Users can perform sequence comparison against all bat-associated virus sequences (nucleic acid or amino acid) in the database or limit their searches within sequences from complete genomes for brevity. The searching output is present in the BLAST result tab in the content panel. Cross-links to the corresponding sequences in GenBank and DBatVir are provided for all hits in the output. The genetic diversity of newly emerged bat-associated viruses is always one of the interesting focuses. For instance, four to five genetically distinct virus strains of henipaviruses have been detected with different virological and biological properties (31). Therefore, to facilitate follow-up sequence analyses, an integrated pipeline for online multiple sequence alignment and phylogenetic tree construction based on the BLAST result is also provided. Users can customize the sequences to be included in follow-up phylogenetic analyses according to their similarities with the query sequence to avoid potential redundancy. The produced multiple alignment and phylogenetic tree can be easily displayed online or downloaded for further offline analyses.

Knowledge of viral hosts enables the identification of maintenance populations from which epidemics may emerge (32). To bridge the gap between virologists and zoologists, the information concerning the bats, such as the common names, diet type and known distribution, as well as the phylogeny of bats are carefully integrated into DBatVir. All bat-related information are presented in a sortable and filterable table, along with example photos of each bat species and useful external links for convenient access to additional information (Figure 1C). Users can use the search entry on the bottom toolbar to quickly extract the information of interested bats by name, diet type or distributed country. Moreover, an intuitive global map with markers indicating the known distributed countries of each bat species is available for users to easily understand the distribution range of the bat. Good knowledge of bats is essential for better interpretation of the data on bat-associated viruses. For example, the incongruent associations between the phylogenies of bats and their SARS-related coronaviruses revealed recent host shifts, which may assist in understanding the emergence of SARS (33). Majority of bats are insectivores, and most of the rest are frugivores. The current data show that the predominant virus families detected in insectivorous and frugivorous bats are *Rhabdoviridae* and *Paramyxoviridae*, respectively. This implies the potential association between bat's diet and the associated virome.

## Discussion

The ability to predict and prevent viral epidemics has become a major objective in the public health disciplines. Effective prediction of future viral zoonoses requires an in-depth understanding of the heterologous viral population in key animal species that will likely serve as reservoir hosts or intermediates during the next viral epidemic (13). The importance of bats as natural hosts for several important viral agents, including rabies virus, Ebola virus, Marburg virus, Hendra virus, Nipah virus and SARS coronavirus, has been established (9–11, 34). Moreover, the past decade has experienced a surge in the discovery of emerging viruses of bat origin, several of which have had a significant impact on public health, tourism and trade. Therefore, it is important to catalog as comprehensively as possible the animal viruses present in bats.

As of January 2014, DBatVir collects information on 4176 bat-associated animal viruses of 23 virus families detected from 196 bat species in 69 countries worldwide. These data will give us an overview of the depth of viral richness observed in bats and provide substantial grist for future attempts to assess and predict epidemic risks. More extensive surveillance in other species of bats and at other geographic locations may be needed to identify more viruses with the potential to cause human diseases or novel viruses related to known human pathogens. To our knowledge, DBatVir is the only publicly available web resource dedicated to bat-associated animal viruses thus far. It devotes to provide comprehensive, up-to-date and well-curated information to the scientific community worldwide. DBatVir will not only be helpful to virologists who want to better understand the virome diversity of bats but will also be useful to zoologists concerned with the health of domestic and wild animals. Furthermore, this database is particularly valuable to epidemiologists and public health researchers, as it is beneficial in the monitoring and tracking of current and future emerging zoonotic diseases.
